# Gut Microbiota-Modulated Metabolomic Profiling Shapes the Etiology and Pathogenesis of Autoimmune Diseases

**DOI:** 10.3390/microorganisms9091930

**Published:** 2021-09-10

**Authors:** Yi-Wen Tsai, Jia-Ling Dong, Yun-Jie Jian, Shin-Huei Fu, Ming-Wei Chien, Yu-Wen Liu, Chao-Yuan Hsu, Huey-Kang Sytwu

**Affiliations:** 1 Department of Family Medicine, Chang Gung Memorial Hospital, Keelung, No.222, Maijin Road, Keelung 204, Taiwan; tsaiyiwen@gmail.com; 2 College of Medicine, Chang-Gung University, No.259, Wenhua 1st Road, Guishan Dist., Taoyuan City 333, Taiwan; 3 Graduate Institute of Medical Sciences, National Defense Medical Center, No.161, Section 6, Min Chuan East Road, Neihu, Taipei 114, Taiwan; 4 Department and Graduate Institute of Microbiology and Immunology, National Defense Medical Center, No.161, Section 6, Min Chuan East Road, Neihu, Taipei 114, Taiwan; irenejld00@gmail.com (J.-L.D.); carol21618@gmail.com (Y.-J.J.); winniefold@gmail.com (S.-H.F.); pantherchien@gmail.com (M.-W.C.); 5 National Institute of Infectious Diseases and Vaccinology, National Health Research Institutes, No.35, Keyan Road, Zhunan, Miaoli 350, Taiwan; candy_77615@yahoo.com.tw; 6 Graduate Institute of Life Sciences, National Defense Medical Center, No.161, Section 6, Min Chuan East Road, Neihu, Taipei 114, Taiwan; 7 Molecular Cell Biology, Taiwan International Graduate Program, Academia Sinica, No.128, Academia Road, Section 2, Nankang, Taipei 115, Taiwan

**Keywords:** intestinal microbiome, metabolomic profiling, autoimmunity, type 1 diabetes, multiple sclerosis, rheumatoid arthritis

## Abstract

Autoimmunity is a complex and multifaceted process that contributes to widespread functional decline that affects multiple organs and tissues. The pandemic of autoimmune diseases, which are a global health concern, augments in both the prevalence and incidence of autoimmune diseases, including type 1 diabetes, multiple sclerosis, and rheumatoid arthritis. The development of autoimmune diseases is phenotypically associated with gut microbiota-modulated features at the molecular and cellular levels. The etiology and pathogenesis of autoimmune diseases comprise the alterations of immune systems with the innate and adaptive immune cell infiltration into specific organs and the augmented production of proinflammatory cytokines stimulated by commensal microbiota. However, the relative importance and mechanistic interrelationships between the gut microbial community and the immune system during progression of autoimmune diseases are still not well understood. In this review, we describe studies on the profiling of gut microbial signatures for the modulation of immunological homeostasis in multiple inflammatory diseases, elucidate their critical roles in the etiology and pathogenesis of autoimmune diseases, and discuss the implications of these findings for these disorders. Targeting intestinal microbiome and its metabolomic associations with the phenotype of autoimmunity will enable the progress of developing new therapeutic strategies to counteract microorganism-related immune dysfunction in these autoimmune diseases.

## 1. Introduction

### 1.1. The Pathophysiology of T Cell-Modulated Autoimmune Disease

T cell-modulated autoimmune diseases, including type 1 diabetes (T1D), multiple sclerosis (MS), and rheumatoid arthritis (RA), are chronic inflammatory disorders in specific organs and contribute to critical clinical problems because of their prevalence in young populations and the associated healthcare cost. The prevalence and incidence of these autoimmune diseases have increased in both developed and developing countries in the last 30 years [1,2,3]. These organ-specific diseases result from the disorders of the immune system induced by self-antigens. In addition, environmental factors have been reported to regulate and modulate the development of such diseases both in humans and mouse models. The activation of T cells by self-antigens in specific organs leads to the expression of inflammatory cytokines and damage of the target tissues [4]. Moreover, differential modulation of environment and lifestyle is regarded as the principal contributor to the enhanced prevalence and pathogenesis of these chronic immune-mediated diseases [5,6].

### 1.2. The Effect of Microbiota on the Development of Autoimmune Diseases

Individuals’ microbiomes can be fingerprinted by the rare microbial strains, whereas the unique form of microbial fingerprinting from personal microbiome provides the different functions to deliver valuable clues about the past exposures to environmental influences in inflammatory processes [7]. It has been reported that personalized microbiome dynamics dissected by cytometric fingerprints can provide the rapid progress to identify microbiome-associated inflammatory diseases and calculate the difference in the community composition of microbiome to evaluate the inflammatory level of autoimmune diseases in both murine models and human, and therefore can serve as the potential diagnostic tool to quantify the diversity of microbiome [8,9,10]. Moreover, the molecular dissection for gut microbial metabolism of ingested compounds will provide personalized medicine and inform toxicology risk assessment to modulate the drug discovery to affect the regenerative development [11]. Recent studies have revealed that modulation of bacterial populations with metabolic properties is critical for the association with inflammatory processes [12]. The short-chain fatty acids (SCFA) generated from the microbiota-accessible carbohydrates in dietary fiber play crucial roles for maintaining gut barrier homeostasis [13]. Dietary fiber-mediated short-term intervention is able to modulate the metabolic function and the bacterial community structure in the intestinal tissues [14]. Moreover, the intervention with the high-fiber diet or the diet with fermented foods have the potential to promote the microbial diversity and downregulate the expression markers for immune-modulated inflammatory progress [15]. These results suggest that the dietary interventions act on both the microbiome and the immune system and may be a critical strategy for alleviating the impact of the industrial microbiome on the inflammatory progress of autoimmune diseases.

### 1.3. The Interaction of Microbiota and the Immune System

**Figure 1:**
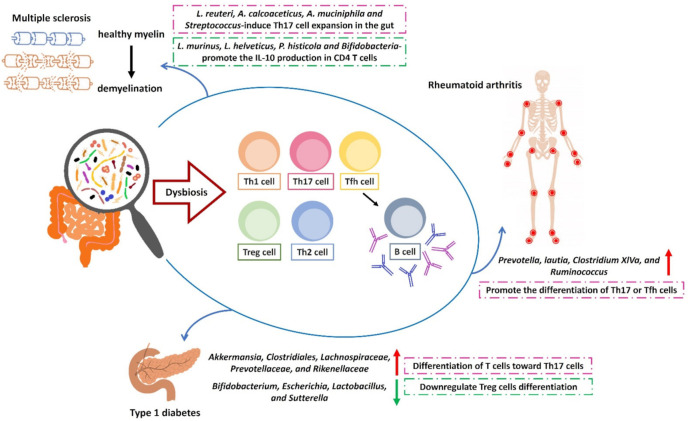
Microbiota modulates the differentiation T helper cells in the pathogenic conditions of autoimmune diseases. The critical interaction between gut microbiota and immune cells in autoimmune diseases. The outcome of gut microbiota-mediated inflammation by the different subsets of CD4^+^ cells in the specific tissue. In T1D, the augmented expression of bacteria from *Akkermansia*, *Clostridiales*, *Lachnospiraceae*, *Prevotellaceae*, and *Rikenellaceae* in the gut can promote the Th17 cell differentiation, whereas the decrease of *Bifidobacterium*, *Escherichia*, *Lactobacillus*, and *Sutterella* contribute to the downregulated expression of Treg cells. In MS, the enhanced expressions of *L. reuteri*, *A. calcoaceticus*, *A. muciniphila*, *Streptococcus* modulate the expansion of intestinal Th17 cells, whereas the upregulation of *L. murinus*, *L. helveticus*, *P. histicola* and *Bifidobacteria* is able to augment the expression of the anti-inflammatory cytokine IL-10 in Th cells. In RA, bacteria of *Prevotella*, *Lautia*, *Clostridium XIVa*, and *Ruminococcus* can promote the differentiation of Th17 or Tfh cells.

The microbiota is critical for the balance of the host immune system, whereas the composition of microbiota provides multiple modulatory functions to the immune system such as synthesizing nutrients and regulating immune responses to antigens [16,17]. Dysbiosis of gut microbiome is highly associated with the damages of specific tissues and inflammatory progresses in susceptible individuals [18]. During the development of autoimmune diseases, the changes of microbiome are critical for modulating inflammation and promoting the loss of immune tolerance [19,20]. Previous studies reported that that T helper (Th) cells are critical modulators of the inflammation of autoimmune diseases by a range of pathogens, and the modulation of cytokines can differentiate into multiple lineages of Th cells with distinct effector subsets, including Th1, Th2, Th17, and regulatory T (Treg) cells [21,22]. The collective functional capacity and diversity maintenance of the microbiota plays the important roles in promoting optimal metabolic regulation for the development of Th1, Th2, Th17, and Treg cells in the immune system. It has been reported that the genus *Clostridium* clusters IV and XIVa can provide an environment rich in transforming growth factor-β to promote the development of Treg cells and modulate the intestinal homeostasis by preventing inflammation [23]. Moreover, Toll-like receptors 2 (TLR2) and CD39 on T cells are critical for the recognition of microbial patterns and are required for Treg function during the inflammatory progress [24,25], revealing that the immune system is able to classify between pathogens and the microbiota through recognition of symbiotic bacterial molecules (Figure 1).

Studies on the roles of commensal microorganisms as immune modulators have shown that the presence of commensal bacteria-derived signals is critical for regulating the immune system development and the host immune response [26,27]. Recent studies have revealed that the initial stages of the pathological immune response associated with autoimmune diseases occur at the sites of mucosal tissues, such as the intestinal or oral mucosa, and are highly associated with the abundance of specific bacterial species [28,29]. In this paper, we will review the modulatory effects of host microbiota on T cell-mediated autoimmune diseases, including T1D, MS, and RA (Figure 1).

## 2. Overview of the Modulatory Effects of Host Microbiota on Autoimmune Diseases

### 2.1. Type 1 Diabetes

#### 2.1.1. The Introduction of T1D and Gut Microbiota

T1D is a T cell-modulated autoimmune disease caused by the damage of β-cells in pancreatic islets, and patients with T1D need lifelong exogenous insulin treatment [30]. The pathogenesis of T1D depends on the cytolytic function of islet antigen-specific CD4^+^ and CD8^+^ T cells [31]. In addition, innate immune cells, such as nature killer cells and dendritic cells (DCs), are involved in regulating the onset of autoimmunity and the development of T1D [32,33]. Moreover, recent reports revealed that the pathogenesis of T1D is complex and contributes to interactions between the genetic and environmental determinants, such as the composition of gut microbiota, which is the indispensable element to the rapid increase of T1D [34]. Furthermore, the immune system closely interacts with the gut microbial community, which plays important roles in shaping the immunity by educating the immune cells and enabling their functional maturation [35].

The phenotypes of gut microbiota in humans are influenced by differences in geography (rural vs. urban) and lifestyle (westernized vs. nonwesternized) [36]. Kemppainen et al., demonstrated that gut microbiome in children showed strong physical differences in subjects at high risk of T1D [37]. The collaboration of a European childhood diabetes registers (EURODIAB) study, which assessed the incidence of newly diagnosed T1D in children aged <15 years, revealed that Finland had the highest incidence of T1D compared with other countries [38]. The study of the Environmental Determinants of Diabetes in the Young (TEDDY) also reported that the early gut microbial composition was correlated with human leukocyte antigen (HLA)-conferred susceptibility to T1D and the bacterial diversity in children was lesser in Finland than in other locations [37,39]. Tommi et al., analyzed 10,913 metagenomes in fecal samples from 783 mostly white, non-Hispanic children. They used samples that were collected in the TEDDY study to clarify the association between microbiome, development of T1D, and the early-life use of antibiotics. In the control children, the microbiomes contained more genes that were associated with fermentation and the biosynthesis of SCFAs. Three *Bifidobacterium* species (*B. bifidum*, *B. breve*, and *B. longum*) were found to be dominant in the first year of life, and a subset of *B. longum* was specifically present in breast-fed infants [40]. It has been reported that human milk oligosaccharides regulate the microbiota via the modulation of gut microbial metabolites and direct priming of the response of Treg cells in autoimmune systems. Moreover, the protective role of SCFAs derived from human milk oligosaccharides against the development of T1D was observed in both humans and rodent models [40,41]. These findings suggest that gut microbiota has a potential role in modulating the development of T1D.

**Figure 2:**
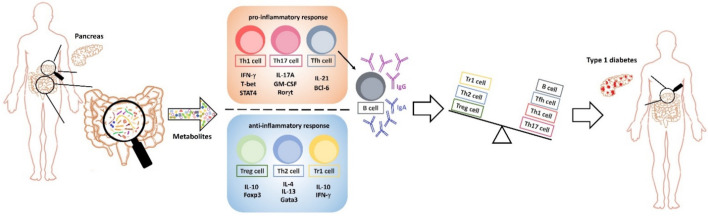
Gut microbiota-modulated regulation of the damage of pancreatic tissues by adaptive immune cells. The production of cytokines by distinct T helper cells are indicated in components involved in regulating Th1, Th2, Th17, Tfh, Treg, and B cells. These pro-inflammatory cytokine-producing Th subsets include T-bet/STAT4-mediated Th1 (interferon (IFN)-γ), Rorγt-modulated Th17 (IL-17A, GM-CSF), and BCL-6-mediated Tfh (IL-21), whereas these anti-inflammatory cytokine-secreting Th subsets are Gata3-modulated Th2 (IL-4, IL-13), Foxp3-mediated Treg (IL-10), and Tr1 (IL-10, IFN-γ) cells. The networks of cytokines in pancreatic tissues affected by gut microbiota regulate the development of adaptive immune cells.

The commensal microbiome in humans has markedly changed in the past seven decades because of modified living conditions with consumption of processed water and food and overuse of antibiotics in agriculture and medical treatment. The dramatically changed microbiome and the associated metabolomic profile play critical roles in the adaption of human gut microbiota [42,43]. In adult gut microbiota, *Bacteroidetes* (Gram-negative bacteria) and *Firmicutes* (Gram-positive bacteria) are the two main phyla, while *Verrucomicrobia*, *Proteobacteria*, and *Actinobacteria* are normally minor constituents of the human gut microbiota [44]. Microbiome equilibrium in the gastrointestinal tract appears to be essential for hindering strong inflammatory responses to maintain homeostasis in the host [45,46]. Overgrowth of certain microorganisms and loss of others might contribute to an imbalance of the gut microbial ecosystem, which is called dysbiosis. Moreover, dysbiosis of microbiota contributes to chronic inflammation and the development of T1D [47] and inflammatory bowel disease [48]. These findings suggest that gut microbiota has a potential role in modulating the progress of T1D (Figure 2).

#### 2.1.2. The Modulatory Effect of Gut Microbiota in T1D

The gut microbiota has important effects on both mucosal and systemic immune systems of the hosts in rodent models, especially the nonobese diabetic (NOD) mice, which is considered the most common animal model to study the complex mechanisms of genetic and immunological tolerance in clinical diabetes [31]. The interaction between intestinal commensal bacteria and innate immunity is regarded as an important epigenetic factor that contributes to the susceptibility for T1D. Moreover, the detection of pathogen-associated molecular patterns and regulation of host immune responses are modulated by toll-like receptors (TLRs) [49,50]. Myeloid differentiation factor 88 (MyD88) is an adaptor protein involved in signaling by multiple TLRs and interleukin (IL)-1 receptor. To clarify the role of TLRs in the development of T1D, NOD mice with genetic deficiency in MyD88 (NOD.*Myd88*^−/−^) were generated and reared under specific pathogen-free (SPF) or germ-free (GF) conditions [51]. Under the SPF conditions, NOD.*Myd88*^−/−^ mice were fully protected from T1D. Antibiotic-treated SPF NOD.*Myd88*^−/−^ mice showed a higher incidence of T1D than untreated mice. Surprisingly, NOD.*Myd88*^−/−^ mice developed diabetes under the GF conditions. Moreover, the GF mice with altered Schaedler flora were resistant to the development of T1D [52]. Furthermore, the deficiency of MyD88 in NOD mice contributed to altered composition of intestinal microbiota and strongly m CD8^+^ T cell-mediated T1D development through gut microbiota in islet-specific glucose-6-phosphatase catalytic subunit-related protein (IGRP)-reactive CD8^+^ T cell receptor NY8.3 transgenic NOD mice [51,53]. In addition, a microbial peptide mimic (derived from a magnesium transporter (GenePept accession No. WP_006806773) of *Leptotrichia* *goodfellowii*) of *Fusobacteria* and the bacteria directly activated IGRP-specific NY8.3 T cells and accelerated the development of diabetes [54]. Interestingly, a gut microbial mimic expressed by a species of the genus *Bacteroides* that encodes a low-avidity mimotope of IGRP206-214 was demonstrated to regulate the recruitment of diabetogenic CD8^+^ T cells to the gut and suppress the development of colitis through targeting the gut DCs [55]. In addition to MyD88, toll-IL receptor-domain-containing adapter-inducing interferon-β (TRIF) is a critical adaptor protein downstream of TLR signaling, especially TLR3 and TLR4. The deficiency of TRIF protected NOD mice from autoimmune diabetes only when housed with wild-type NOD mice. The abundance of *Sutterella* (*Proteobacteria*) and *Rikenella* (*Bacteroidetes*) was significantly reduced in the NOD mice with the deficiency of TRIF [56]. Moreover, administration of human gut microbiota to germ-free (GF) NOD mice can modulate their development of autoimmune diabetes, but the pace of function loss of β-cell loss was not transferable to the rodent model [40]. These findings suggest that the interactions between innate immunity and gut microbiota were involved in the development of T1D. Furthermore, colonization of the gut with Gram-positive aerobic rods (*Bacillus cereus*) [57] or segmented filamentous bacteria was found to attenuate the development of T1D. Compared with untreated control mice, the induction of Treg cells was slower in the small intestinal lamina propria (siLP) of these mice [58]. In addition to colonization studies, antibiotic treatment studies are used to evaluate the role of intestinal microbiota in the disease progression of T1D.

The use of antibiotics in agriculture and medical treatment has increased over the past 50 years. Moreover, antibiotic use is involved in the increased incidence of some diseases, such as obesity, and *Clostridium difficile* infections [59]. Increasing evidence indicates that environmental factors that alter the composition of gut microbiota strongly impact the risk of developing T1D [60,61,62]. Wild-type neonatal NOD mice treated with vancomycin (a glycopeptide antibiotic that inhibits cell wall synthesis by targeting Gram-positive bacteria) from birth until weaning (4 weeks) showed slower onset and lower incidence of diabetes compared with the untreated group. The populations of CD4^+^ T cells in the siLP and proinflammatory cytokines, such as interferon-γ and tumor necrosis factor-α, were higher in neonatally vancomycin-treated mice. Moreover, the number of IL-17-producing T cells was higher in neonatally vancomycin-treated mice than in adult vancomycin-treated mice and untreated mice. Furthermore, the composition of intestinal microbiota was analyzed in the neonatally vancomycin-treated mice using pyrosequencing. The major phyla of *Firmicutes* and *Bacteroidetes* were found to be depleted in the gut, while *Akkermansia muciniphila* became a dominant species in the gut of these mice. *A.* *muciniphila* might play a protective role in the early stage of development of autoimmune diabetes [63]. Bacteria of the genera *Escherichia*, *Lactobacillus*, and *Sutterella* were found to be increased in the gut of antibiotic-treated mice, while those of *Clostridiales*, *Lachnospiraceae*, *Prevotellaceae*, and *Rikenellaceae* were reduced [53,63]. However, different antibiotic treatments targeting distinct bacteria contribute to opposite effects in autoimmune disease development. Offspring of vancomycin-treated pregnant female mice showed accelerated autoimmune diabetes development, while neomycin-treated offspring showed attenuation of diabetes, and the composition of the gut microbiota was distinctly different than that in untreated control mice [64].

Antibiotic use in early life changes the gut microbiome and results in a predisposition to disease. Pulsed therapeutic antibiotics (PTA) treatment in early life accelerated the incidence of T1D in male NOD mice. Moreover, the microbiota composition and structure was altered in these mice compared with control mice. *Bifidobacterium* (including *B. adolescentis*, *B. animalis*, and *B. pseudolongum*) levels were lower and *Akkermansia* levels were higher in PTA-treated male mice. Th17 and Treg populations and intestinal serum amyloid A (SAA) expression were lower in prediabetic male PTA-treated mice than in control mice. Microbial lipid metabolism and the expression of cholesterol biosynthetic genes in the host were affected by PTA [65]. *Bacteroides fragilis* and *B. fragilis*-like commensals cause proinflammatory responses and lead to T1D in at-risk subjects [66].

#### 2.1.3. Targeted Therapies for Gut Microbiota in Autoimmune Diabetes

It has been reported that both environmental factors and genetic risks play important roles in the development of T1D [67,68]. The intestinal microbiota composition is different between C57BL/6 and NOD mice because of distinct genetic backgrounds. *Lactobacillus* was the dominant bacteria in NOD mice, while *Allobaculum* was in C57BL/6 mice. Early-life exposure and housing conditions affect the microbiome composition in NOD mice, while the genetic background of NOD mice constrains the overall microbiota community composition. Early-life factors, including breastfeeding and exposure to being preconception (in utero and postnatally), are related to the increased risk of T1D [69]. NOD.insulin-dependent diabetes (Idd)3/Idd5 mice, which have both protective alleles with the *Idd3* locus (*Il2*) and the *Idd5* locus (*Ctla4*, *Slc11a1*, and *Acadl*), were protected from T1D and possessed dramatic alterations of microbiota composition compared with wild-type mice [51,52]. Decreased inflammation in the ileum and colon and production of antimicrobial peptides (AMPs) were observed in NOD.Idd3/Idd5 mice, while mucous production by goblet cells and levels of regulatory cytokine IL-10 were higher in these mice than in control mice [70,71]. Administration of IL-2 therapy reduced inflammation, increased the population of Treg cells, and changed the microbiota in NOD mice. Bacteria belonging to *Bacteroidales* and *Oscillospira* were significantly reduced and *Bifidobacteria* were increased following IL-2 therapy. Moreover, participants in the TwinsUK cohort who were at high risk of T1D and had IL-2 pathway loci showed some similar microbiome alterations as those observed in the rodent model [72].

Accumulated evidence suggests that microbial-derived metabolites influence the immune response [73] and might result in the development of T1D. SCFAs are the major metabolites of the gut microbiota and are mostly produced in the colon via the bacterial fermentation of dietary fiber. The function and levels of induced Treg cells in the colon are stimulated by SCFAs. Treatment of NOD mice with SCFAs reduced immunoglobulin a response, which is induced by the gut bacteria, and dampened the severity of insulitis [74]. Apart from SCFAs, acetate and butyrate also affect the immune response [75]. Feeding specialized diets, which included acetate or butyrate, to NOD mice provided a protective effect against T1D. A diet containing acetate decreased the population of autoreactive T cells in lymphoid organs, while a diet containing butyrate enhanced the development of Treg cells [76]. AMPs play a critical role in eliminating infection and regulating the intestinal microbiota. Serum levels of cathelicidin, which is an AMP, were reduced in patients with T1D compared with healthy subjects [77]. Cathelicidin-related antimicrobial peptide (CRAMP), which was produced by insulin-secreting β-cells and the production of CRAMP, were defective in NOD mice. Treatment of prediabetic NOD mice with CRAMP decreased the incidence of autoimmune disease and induced regulatory immune cells (DCs and T cells) in pancreatic islets. In addition, the levels of CRAMP produced by β-cells were regulated by SCFAs that were produced by the gut microbiota [78]. Another AMP, mouse β-defensin 14, whose expression is induced by pancreatic innate lymphoid cells, attenuated the development of T1D in NOD mice. In addition, pancreatic innate lymphoid cells regulate the IL-22 secretion induced by intestinal microbiota-derived metabolites [79]. These findings suggest that the metabolites of gut microbiota provide a helpful and natural approach against the several immunological defects that lead to T1D.

Recent research has addressed the role of the gut microbiome in autoimmune diseases [80,81,82]. Longitudinal studies on humans revealed that the diversity and intestinal dysbiosis of microbiota, which is regarded as a group of beneficial microorganisms, were decreased in patients with T1D. Moreover, bacteria of the genus *Bacteroides* were increased and *B.* *adolescentis* and *B.* *pseudocatenulatum* were decreased in children with β-cell autoimmunity [83]. Oral administration of *Lactobacillus johnsonii* strain N6.2 in the rodent model was observed to a Th17 cell bias in the mesenteric lymph nodes and dampened the onset of T1D [84]. However, the mechanisms by which the alterations of microbiota modulate tissue-specific autoimmune responses are still not well understood.

### 2.2. Multiple Sclerosis

#### 2.2.1. The Role of Gut Microbiota in Central Nervous System-Based Autoimmune Diseases

MS is an inflammatory demyelinating disease which involves the immune system and CNS interaction. Therefore, animal modeling has been critical for addressing MS pathogenesis [85,86,87]. The three most characterized animal models of MS are: (1) experimental autoimmune encephalomyelitis, which is the widest research bench of studying MS through immunization with self-antigens to induce autoimmunity; (2) virus-induced demyelinating disease, the demyelination mice induced by viral infections. The best studied is Theiler’s murine encephalomyelitis virus (TMEV) [88]; (3) toxic models of demyelination and remyelination [89].

Immune cells, such as DCs and Th cells, are critically involved in attacking the myelin sheath to cause myelin loss and neuroaxonal degeneration to disrupt neuronal signaling [90]. DCs present the myelin epitopes to myelin-reactive T cells and stimulate them to differentiate into Th1 and Th17 cells. These Th cells are reactivated by CNS-resting tissue macrophages (microglia) to contribute to brain inflammation and myelin damage through the expression of inflammatory cytokines, including interferon-γ, IL-17, and granulocyte-macrophage colony-stimulating factor [91,92,93,94]. The development of MS is strongly associated with environmental factors, such as diet-modulated microbiota, which promote the interaction between DCs and T cells to modulate the risk of disease [95]. Furthermore, GF mice are resistant to the progress of EAE development compared with SPF mice, and reduced gut commensal microflora by oral antibiotic treatment also regulates the susceptibility to EAE [96,97]. Moreover, metagenomic and metabolomic analyses indicated that gut microbiota-derived metabolites modulate the pathogenesis of MS by affecting brain function and behavior through regulating the activation of T cells and their cytokine production [98,99]. These findings indicate that the gut microbiota has crucial roles in influencing the pathogenesis of EAE and MS.

#### 2.2.2. The Modulatory Effect of Gut Microbiota on the Diet–Microbiota Axis in MS and EAE

The gut microenvironment is critical for the activation and proliferation of myelin-reactive Th17 cells at the initiation stage of EAE, and then these cells migrate to the CNS to cause nerve inflammation during the development of EAE [100]. Moreover, the *Streptococcus* and *Akkermansia* was the expansion of Th17 cells and the *Prevotella* was promoted IL-10 producing in the intestinal tissue, and the phenomena was highly correlated with the severity of disease [101]. Patients with MS have a higher proportion of *Streptococcus* and a lower proportion of *Prevotella* in the small intestine than healthy controls [101]. Recent studies have revealed that, aside from CD4^+^ T cells, microbiota is also crucial for the maturation and function of microglia and astrocytes, which are involved in the pathogenesis of MS [102,103]. These findings suggest that the microbiota-modulated effect on the adaptive immune response is critical for the pathogenesis of MS.

Certain environmental factors, such as dietary habits, are strongly associated with human health and known to regulate the risk for MS and EAE. The severity and development of EAE were augmented in mice fed with a typical Western diet, which contains abundant salt and saturated fat [104]. Moreover, a decrease in *Lactobacillus* levels was observed during the development of EAE, and this finding may contribute to the impaired composition of the gut microbiota [105]. Moreover, dietary habits play critical roles in affecting the composition and function of microbes in the gut [13]. The levels of *Lactobacillus murinus* were decreased in the gut of mice with the feeding of the high-salt diet compared with that of mice with the feeding of the normal salt diet, and the oral supplementation of *L. murinus* was able to reduce the population of Th17 cells and ameliorate the progress of EAE during feeding of the high-salt diet [106]. Furthermore, the level of sodium intake was positively correlated with the clinical disease activity in patients with MS [107]. Apart from *L. murinus*, the administration of *Lactobacillus helveticus* SBT2171 (LH2171) also attenuated EAE and reduced the production of IL-6 to impair the differentiation of Th17 cells [108]. However, 16S ribosomal RNA gene sequencing revealed that the levels of *L. murinus* and *Lactobacillus reuteri* exhibit an inverse correlation in the microbiome during the progress of EAE, implying that *L. reuteri* may contribute to the pathogenesis of EAE [109]. A recent study also reported that the activation of myelin-specific T cells is modulated by *L. reuteri*, which potentially mimics myelin oligodendrocyte glycoprotein peptides in the small intestine [110]. The pathogenicity of myelin-specific T cells is enhanced by operational taxonomic unit 0002 to augment the development of EAE [110]. These findings suggest that the different species of *Lactobacillus* possess distinct and critical roles in the development of EAE and contribute to the modulatory effect on the microbiota–gut–brain axis.

Dr. Emanuel Vamanu and his colleague had reported that the modulation of microbiota in the degenerative diseases is critical for the alleviations of neurodegenerative pathologies [111]. The composition of gut microbiota was altered by dietary habits [112]. Obese patients have increased Firmicutes/Bacteroidetes ratio in the fecal microbiota [113,114]. The obese mice also showed an increased Firmicutes and decreased Bacteroidetes in feces [115]. Furthermore, HFD-induced obese mice developed an exacerbated EAE and promote CNS infiltration through IL-6 and CCL-2 [116]. In the US, the researchers found that adolescents of women with a body mass index (BMI) ≥30 kg/m^2^ had a 2-fold increased risk of MS [117]. The study of Sweden found that obesity at age 20 (BMI ≥ 27 kg/m^2^) in both men and women was associated with a greater than 2-fold increased risk of MS [118]. Additional research also found a 2-fold increased risk of MS as a result of obesity, including data from Norway and Italy [119]. These studies suggest the link between obesity, gut microbiota, and the pathogenesis of MS.

#### 2.2.3. Targeted Therapies for Gut Microbiota in MS and EAE

Fatty acids are classified as long-chain fatty acids (LCFAs) and SCFAs. LCFAs are highly abundant in western diets. SCFAs are metabolized by the gut microbiota. Mice fed with LCFAs developed more severe EAE via expansion of pathogenic Th17 cells in the small intestine, whereas those fed with SCFAs exhibited attenuated development of EAE through the promotion of Treg cell differentiation [120]. Moreover, SCFAs, including acetate, propionate, and butyrate, are the major products of the microbiota fermentation of dietary fiber in the intestines and have a critical role in the microbiota–gut–brain axis [121,122]. The oral administration of acetates, such as glatiramer acetate and oleanolic acid acetate, has been reported to alleviate clinical symptoms of EAE [123,124]. Furthermore, the oral administration of butyrate in mice suppressed demyelination via the accumulation and maturation of microglia, leading to enhanced remyelination in demyelinated lesions [125]. The levels of SCFAs, including acetate, propionate, and butyrate, are decreased in patients with MS compared with healthy controls, and SCFAs can induce IL-10 production by Treg cells [126]. In addition, dietary restriction of tryptophan, which is an essential amino acid, abrogated the clinical signs of EAE through inhibition of IL-17A and the granulocyte-macrophage colony-stimulating factor, but promoted IL-10 secretion leading to impaired encephalitogenicity of T cells [127]. Thus, diet and dietary supplementation are the major factors that alter the composition of the gut microbiota.

**Figure 3:**
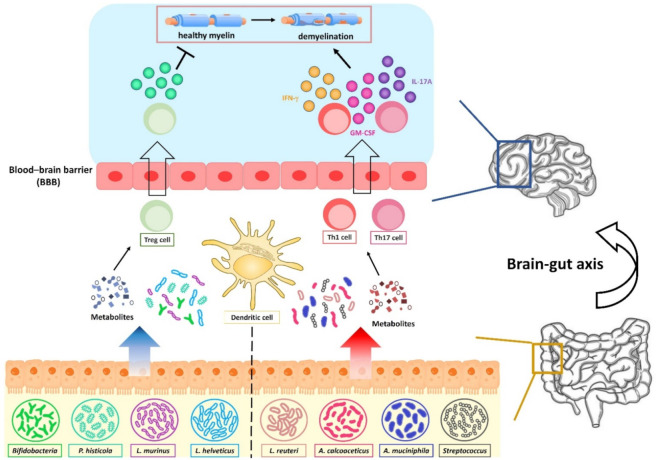
Fecal microbiota regulates the polarity of T helper cells in the brain–gut axis. The enhanced expressions of *L. reuteri*, *A. calcoaceticus*, *A. muciniphila*, *Streptococcus* modulate the expansion of intestinal Th1 and Th17 cells to promote the expression of IFN-γ, IL-17A, and GM-CSF, whereas the upregulation of *L. murinus*, *L. helveticus*, *P. histicola* and *Bifidobacteria* is able to augment the expression of the anti-inflammatory cytokine IL-10 from Treg cells to modulate the barrier function. Gut microbiota-modulated metabolomic profiling regulates the chronic inflammation in the gut and promotes the pathogenic T helper cell infiltration into the brain.

Fecal microbiota transplantation is a treatment strategy that transfers fecal microbiota from a donor to a recipient [128]. In mice with EAE that received fecal microbiota enriched with *A.* *muciniphila* and *Acinetobacter calcoaceticus* from patients with MS showed more severe disease and decreased production of IL-10 from CD4^+^ T cells [129]. *Prevotella histicola*, which is abundant in human feces after the consumption of a high-fiber diet, results in attenuating the development of EAE through downregulation of Th17-associated cytokine production and upregulation of Treg cell expression [130]. Only two cases have been reported regarding the effect of fecal microbiota transplantation on MS progression. In both cases, amelioration of the disease was observed [131,132]. Moreover, microRNAs (miRNAs) in the feces are involved in the pathogenesis of MS and EAE [133]. miRNA sequencing using a next-generation sequencing platform indicated that the expression of miRNAs is changed in motor neurons during the development of EAE, and the miRNA expression profile is correlated with the clinical symptoms of EAE [134]. In addition, levels of the miRNA miR-30d-5p are increased in the feces of both patients with EAE at the disease peak and patients with MS. The oral administration of miR-30d-5p ameliorated EAE and promoted Treg cell expression through the increased abundance of commensal microbiota, such as *A. muciniphila*, in the gut [135]. Therefore, dietary modification may prevent or attenuate the development of EAE/MS via maintenance of intestinal homeostasis (Figure 3).

### 2.3. Rheumatoid Arthritis

#### 2.3.1. The Introduction of Microbiota and RA

Rheumatoid Arthritis is a systemic autoimmune disease with the chronic synovial inflammation, hyperplasia, and immune cell infiltration in multiple joints, which eventually leads to the degradation and damage of the cartilage, bone erosion, and polyarthritis [136]. RA affects nearly 1% of the population worldwide [137], and the comorbidities related to RA often lead to high morbidity and reduced life expectancy [138]. The etiology of RA is multifactorial, including genetic and environmental factors as well as immune-mediated synovial inflammation and cytokine production [139]. The environmental risk factors known to trigger the development of RA in genetically susceptible individuals include tobacco smoking [140], diet, and the mucosal commensal microbiota [141,142]. Increasing evidence has highlighted the importance of the altered gut microbiome in the pathogenesis of RA [143,144,145].

#### 2.3.2. The Role of Intestinal Microbiota in Murine Models of Arthritis

Dysbiosis of the gut microbiota is related to the development of arthritis in several different mouse models. For example, van den Berg et al., elucidated that spontaneous onset of arthritis was abrogated in IL-1 receptor antagonist-knockout (*IL1rn*^−/−^) mice, which is an autoimmune T cell-modulated arthritis model, housed under GF conditions [116]. Nevertheless, arthritis development was induced on monocontamination of GF *IL1rn*^−/−^ mice with *Lactobacillus bifidus* [146]. Abdollahi-Roodsaz et al., demonstrated an expansion of Th17 cells in the LP and increase of IL-17 production by intestinal LP lymphocytes in the autoimmune arthritis-prone *IL1rn*^−/−^ mice, and these effects were transferable to wild-type mice by fecal microbiota transplantation. In addition, the onset and severity of arthritis could be attenuated in *IL1rn*^−/−^ mice housed under GF conditions or treated with selective antibiotics [145].

Notably, naïve SKG mice, which are zeta-chain-associated protein kinase 70 (Zap70) gene point mutation arthritis-prone mice [144], do not develop arthritis under GF conditions [147]. Nevertheless, the severity of ankle arthritis was higher in SKG mice with the breeding in the SPF conditions than those housed under GF conditions after treatment with curdlan, which is a proinflammatory pathogen-associated molecular trigger. Takeda et al., showed that dysbiosis via inoculation of fecal samples from patients with RA into GF SKG mice could induce the development of severe arthritis through activation of autoreactive T cells and an increased number of Th17 cells in the murine intestine compared with SKG mice inoculated with fecal microbiota from healthy controls [143].

In addition, Kuhn et al., demonstrated that depletion of intestinal microbiota could reduce disease severity in the collagen-induced arthritis mouse model. Moreover, reduced levels of inflammatory cytokines, such as IL-17A and IL-22, in the murine intestine and of anti-type II collagen antibodies were observed, which imply that dysbiosis in the murine gut microbiota can modulate the mucosal immune responses and affect the development of arthritis in the collagen-induced arthritis mouse model [148]. Nevertheless, further studies are needed to understand which species of the gut microbiota have greater effects on the development of experimental inflammatory arthritis in murine models as well as the direct pathophysiological mechanism underlying these effects. These findings indicate that the gut microbiota plays an important role in the development of arthritis and even one particular species of commensal bacteria is sufficient to induce arthritis in different murine inflammatory arthritis models.

#### 2.3.3. The Role of Intestinal Microbiota in Human RA

Previous studies have demonstrated the robust impact of gut and oral cavity microbiomes on the pathogenesis of RA [149]. Increasing evidence shows alteration in the microbiota composition in patients with RA compared with healthy controls or patients with other diseases. In a systemic review and meta-analysis, decreased levels of *Faecalibacterium* in the gut of patients with early RA and RA compared with healthy controls were found in more than three articles. Moreover, decreased levels of *Streptococcus* and *Haemophilus* and increased levels of *Prevotella* in the oral cavity of patients with early RA and RA compared with healthy controls were also reported in more than three articles, which established a significant difference in the abundance of oral or gut microbiome between patients with RA and healthy controls [150]. In addition, enrichment of *Prevotella* species, especially *Prevotella copri*, in fecal microbiota [29,143,151] and reduced abundance of *Bacteroides* species in the intestine were noted in patients with RA [151,152].

Abnormal abundance of bacterial species was found to be associated with the alteration of lymphocyte subpopulations and cytokine levels, which might contribute to the pathogenesis of RA [153]. Compared with healthy controls, patients with RA had a greater abundance of *Proteobacteria* and lesser levels of *Firmicutes* in the gut microbiota. In patients with RA with lower levels of peripheral subpopulations of T, B, CD4^+^ T, and Treg cells, increased abundance of *Blautia*, *Clostridium XlVa*, and *Ruminococcus* in the gut microbiota was observed. The relative abundances of *Clostridium XlVb*, *Clostridium XVIII*, *Pelagibacterium*, and *Oxalobacter* were correlated with serum cytokine levels in patients with RA. Hence, these findings indicate that the diversity and relative abundance of gut microbiota species of patients with RA were clearly different from those of healthy controls, and different gut microbiota species were closely associated with serum cytokine levels in patients with RA [153].

It has been reported that an augmented number of intestinal Th17 cells and more severe arthritis in SKG mice receiving microbiota from patients with RA and elevated IL-17 cytokines in regional lymph nodes and the colon after treatment with the arthritis-related autoantigen 60S ribosomal protein L23a were found in an in vitro study [143]. Abdollahi-Roodsaz et al., demonstrated that monocontamination of GF mice with *L.* *bifidus* could induce the development of arthritis, and reached the severity scores comparable to those in non-GF mice [146]. In addition, acute stimulation of TLRs, which are primarily contained in the innate immune response to microbial pathogens [50], by a TLR2 agonist, Pam3Cys, could aggravate the severity in *IL1rn*^−/−^ arthritis mice [146]. Moreover, a cross-sectional study conducted in the United Kingdom and Switzerland using genotyping and microbiota data from human blood and stool samples of previous cohort studies demonstrated that *Prevotella* spp. in the gut microbiota are indeed positively correlated with the genotype of RA in the absence of disease onset. This finding suggested that the host genotype is associated with microbiota profile before the development of disease [154]. However, the detailed microbiota-mediated molecular mechanisms involved in the pathological exacerbation of RA in humans need further investigation.

#### 2.3.4. Dietary Modification and Gut Microbiome as a Potential Therapeutic Target for RA

**Figure 4:**
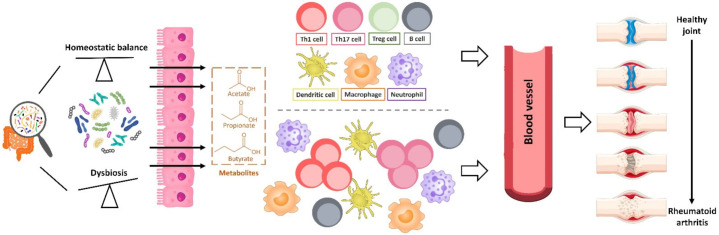
The critical roles of gut microbiota-modulated features in joint-associated colonic inflammation. Microbiota-mediated short-chain fatty acids (SCFA) modulate the risk of RA development and their potential effects on the innate immune cells (Dendritic cell, Macrophage and Neutrophil) or adaptive immune cells (Th1, Th17, Treg, and B cells). The outcome of gut microbiota dysregulation for the chronic inflammation in the joint tissue reflects the balance between pro- and anti-inflammatory mediators, and bacterial component translocation regulates the inflammatory response by enhancing Th1 and Th17 cell differentiation.

Different dietary patterns could influence the composition and function of the gut microbiome [155]. In a nested case-control study, the individuals at risk of RA had lower concentrations of omega-3 fatty acids in red blood cell membranes, which indicated a potential beneficial effect of omega-3 fatty acids on RA-related autoimmunity [156]. In addition, another meta-analysis aiming to explore the association between fish consumption and the subsequent development of RA found a protective effect of fish intake per week [157]. SCFAs produced by the gut flora from ingested plant fibers are known to have various beneficial effects, including improving the function of the gut barrier [158,159] and regulating IgA secretion [160]. Another prospective cohort study conducted by Zaiss et al., showed reduced serum levels of zonulin, which is a biological marker of the intestinal barrier function [161], at the end of the follow-up and an increase in the number of circulating Treg cells, favorable Th1/Th17 ratios, as well as decreased levels of bone erosion markers after 28 days of dietary intervention using fiber supplementation in patients with RA [162]. However, the detailed mechanistic interactions between specific diet components, especially the beneficial effects of SCFAs, and the resulting altered gut microbiome need further investigation. In addition, the pathological autoimmune response triggered in patients with RA initiates at the mucosal sites, such as the intestinal mucosa, instead of initiating in the synovium of the joints [28] and is associated with several particular bacterial species [29]. Thus, modulation of the intestinal microbiome through dietary modification may be a therapeutic target for patients with RA and needs to be investigated (Figure 4).

## 3. Conclusions

This review describes the recent studies that have demonstrated that the development of autoimmune diseases is phenotypically associated with gut microbiota-modulated features at the molecular and cellular levels. The modulatory interactions between the gut microbial community and the immune system are critical for the progression of autoimmune disease, and the future aims, which are to clarify the modulatory effects of these modifications in autoimmune disease may focus on the profiles of gut microbial signatures for the modulation of immunological homeostasis in multiple inflammatory diseases. Taken together, studies on gut microbial community and its associated immune disorders have revealed critical aspects of controlling machinery and may offer new insights into the modulation of immune systems as well as the development of therapeutic treatment targeting the intestinal microbiome and its metabolomic profile rather than using broad immunosuppressive agents. Moreover, targeting intestinal microbiome and its metabolomic associations with the phenotype of autoimmunity will provide a logical motivation for developing new therapeutic strategies to counteract microorganism-related immune dysfunction in these autoimmune diseases.

## Data Availability

Not applicable.

## References

[1] Knip M. (2011). Pathogenesis of Type 1 Diabetes: Implications for Incidence Trends. Horm. Res. Paediatr..

[2] Harjutsalo V., Sund R., Knip M., Groop P.-H. (2013). Incidence of Type 1 Diabetes in Finland. JAMA.

[3] Boyko A., Melnikov M. (2020). Prevalence and Incidence of Multiple Sclerosis in Russian Federation: 30 Years of Studies. Brain Sci..

[4] Davidson A., Diamond B. (2001). Autoimmune Diseases. N. Engl. J. Med..

[5] Okada H., Kuhn C., Feillet H., Bach J.-F. (2010). The ‘hygiene hypothesis’ for autoimmune and allergic diseases: An update. Clin. Exp. Immunol..

[6] Salliot C., Nguyen Y., Boutron-Ruault M.-C., Seror R. (2020). Environment and Lifestyle: Their Influence on the Risk of RA. J. Clin. Med..

[7] Tierney B., Yang Z., Luber J.M., Beaudin M., Wibowo M.C., Baek C., Mehlenbacher E., Patel C.J., Kostic A.D. (2019). The Landscape of Genetic Content in the Gut and Oral Human Microbiome. Cell Host Microbe.

[8] Zimmermann J., Hübschmann T., Schattenberg F., Schumann J., Durek P., Riedel R., Friedrich M., Glauben R., Siegmund B., Radbruch A. (2016). High-resolution microbiota flow cytometry reveals dynamic colitis-associated changes in fecal bacterial composition. Eur. J. Immunol..

[9] Vandeputte D., Kathagen G., D’Hoe K., Vieira-Silva S., Valles-Colomer M., Sabino J., Wang J., Tito R.Y., De Commer L., Darzi Y. (2017). Quantitative microbiome profiling links gut community variation to microbial load. Nat. Cell Biol..

[10] Rubbens P., Props R., Kerckhof F.-M., Boon N., Waegeman W. (2021). Cytometric fingerprints of gut microbiota predict Crohn’s disease state. ISME J..

[11] Koppel N., Rekdal V.M., Balskus E.P. (2017). Chemical transformation of xenobiotics by the human gut microbiota. Science.

[12] Blaser M. (2017). The theory of disappearing microbiota and the epidemics of chronic diseases. Nat. Rev. Immunol..

[13] Makki K., Deehan E.C., Walter J., Bäckhed F. (2018). The Impact of Dietary Fiber on Gut Microbiota in Host Health and Disease. Cell Host Microbe.

[14] Baxter N.T., Schmidt A.W., Venkataraman A., Kim K.S., Waldron C., Schmidt T.M. (2019). Dynamics of Human Gut Microbiota and Short-Chain Fatty Acids in Response to Dietary Interventions with Three Fermentable Fibers. mBio.

[15] Wastyk H.C., Fragiadakis G.K., Perelman D., Dahan D., Merrill B.D., Yu F.B., Topf M., Gonzalez C.G., Van Treuren W., Han S. (2021). Gut-microbiota-targeted diets modulate human immune status. Cell.

[16] Wu H.J., Wu E. (2012). The role of gut microbiota in immune homeostasis and autoimmunity. Gut Microbes.

[17] Belkaid Y., Hand T.W. (2014). Role of the Microbiota in Immunity and Inflammation. Cell.

[18] Khan M.F., Wang H. (2020). Environmental Exposures and Autoimmune Diseases: Contribution of Gut Microbiome. Front. Immunol..

[19] De Luca F., Shoenfeld Y. (2019). The microbiome in autoimmune diseases. Clin. Exp. Immunol..

[20] Crowe W., Allsopp P.J., Watson G.E., Magee P.J., Strain J., Armstrong D.J., Ball E., McSorley E.M. (2017). Mercury as an environmental stimulus in the development of autoimmunity—A systematic review. Autoimmun. Rev..

[21] Imam T., Park S., Kaplan M.H., Olson M.R. (2018). Effector T Helper Cell Subsets in Inflammatory Bowel Diseases. Front. Immunol..

[22] Zhu J., Yamane H., Paul W.E. (2010). Differentiation of Effector CD4 T Cell Populations. Annu. Rev. Immunol..

[23] Atarashi K., Tanoue T., Shima T., Imaoka A., Kuwahara T., Momose Y., Cheng G., Yamasaki S., Saito T., Ohba Y. (2011). Induction of colonic regulatory T cells by indigenous clostridium species. Science.

[24] Round J.L., Lee S.M., Li J., Tran G., Jabri B., Chatila T., Mazmanian S.K. (2011). The Toll-Like Receptor 2 Pathway Establishes Colonization by a Commensal of the Human Microbiota. Science.

[25] Telesford K.M., Yan W., Ochoa-Re J., Pant A., Kircher C., Christy M.A., Begum-Haque S., Kasper D.L., Kasper L.H. (2015). A commensal symbiotic factor derived fromBacteroides fragilispromotes human CD39^+^Foxp3^+^T cells and Tregfunction. Gut Microbes.

[26] Abt M.C., Artis D. (2013). The dynamic influence of commensal bacteria on the immune response to pathogens. Curr. Opin. Microbiol..

[27] Yoo J.Y., Groer M., Dutra S.V.O., Sarkar A., McSkimming D.I. (2020). Gut Microbiota and Immune System Interactions. Microorganisms.

[28] Holers V.M., Demoruelle M.K., Kuhn K.A., Buckner J.H., Robinson W.H., Okamoto Y., Norris J.M., Deane K.D. (2018). Rheumatoid arthritis and the mucosal origins hypothesis: Protection turns to destruction. Nat. Rev. Rheumatol..

[29] Alpizar-Rodriguez D., Lesker T.R., Gronow A., Gilbert B., Raemy E., Lamacchia C., Gabay C., Finckh A., Strowig T. (2019). Prevotella copri in individuals at risk for rheumatoid arthritis. Ann. Rheum. Dis..

[30] Atkinson M.A., Eisenbarth G.S., Michels A.W. (2014). Type 1 diabetes. Lancet.

[31] Anderson M.S., Bluestone J.A. (2005). THE NOD MOUSE: A Model of Immune Dysregulation. Annu. Rev. Immunol..

[32] Turley S., Poirot L., Hattori M., Benoist C., Mathis D. (2003). Physiological β Cell Death Triggers Priming of Self-reactive T Cells by Dendritic Cells in a Type-1 Diabetes Model. J. Exp. Med..

[33] Lehuen A., Diana J., Zaccone P., Cooke A. (2010). Immune cell crosstalk in type 1 diabetes. Nat. Rev. Immunol..

[34] Knip M., Siljander H. (2016). The role of the intestinal microbiota in type 1 diabetes mellitus. Nat. Rev. Endocrinol..

[35] Hooper L.V., Littman D.R., MacPherson A.J. (2012). Interactions Between the Microbiota and the Immune System. Science.

[36] Yatsunenko T., Rey F.E., Manary M.J., Trehan I., Dominguez-Bello M.G., Contreras M., Magris M., Hidalgo G., Baldassano R.N., Anokhin A.P. (2012). Human gut microbiome viewed across age and geography. Nature.

[37] Kemppainen K.M., Ardissone A.N., Davis-Richardson A.G., Fagen J.R., Gano K.A., Leon-Novelo L.G., Vehik K., Casella G., Simell O., Ziegler A.G. (2014). Early Childhood Gut Microbiomes Show Strong Geographic Differences Among Subjects at High Risk for Type 1 Diabetes. Diabetes Care.

[38] Patterson C.C., Dahlquist G.G., Gyürüs E., Green A., Soltész G. (2009). Incidence trends for childhood type 1 diabetes in Europe during 1989–2003 and predicted new cases 2005–2020: A multicentre prospective registration study. Lancet.

[39] The TEDDY Study Group (2007). The Environmental Determinants of Diabetes in the Young (TEDDY) study: Study design. Pediatr. Diabetes.

[40] Vatanen T., Franzosa E.A., Schwager R., Tripathi S., Arthur T.D., Vehik K., Lernmark A., Hagopian W.A., Rewers M.J., She J.-X. (2018). The human gut microbiome in early-onset type 1 diabetes from the TEDDY study. Nature.

[41] Xiao L., Land B.V., Engen P.A., Naqib A., Green S.J., Nato A., Leusink-Muis T., Garssen J., Keshavarzian A., Stahl B. (2018). Human milk oligosaccharides protect against the development of autoimmune diabetes in NOD-mice. Sci. Rep..

[42] Von Hertzen L., Beutler B., Bienenstock J., Blaser M., Cani P.D., Eriksson J., Färkkilä M., Haahtela T., Hanski I., Jenmalm M.C. (2015). Helsinki alert of biodiversity and health. Ann. Med..

[43] Quercia S., Candela M., Giuliani C., Turroni S., Luiselli D., Rampelli S., Brigidi P., Franceschi C., Bacalini M.G., Garagnani P. (2014). From lifetime to evolution: Timescales of human gut microbiota adaptation. Front. Microbiol..

[44] Lozupone C.A., Stombaugh J.I., Gordon J.I., Jansson J.K., Knight R. (2012). Diversity, stability and resilience of the human gut microbiota. Nature.

[45] Renz H., Brandtzaeg P., Hornef M.W. (2011). The impact of perinatal immune development on mucosal homeostasis and chronic inflammation. Nat. Rev. Immunol..

[46] Sommer F., Bäckhed F. (2013). The gut microbiota—Masters of host development and physiology. Nat. Rev. Microbiol..

[47] Dunne J.L., Triplett E.W., Gevers D., Xavier R., Insel R., Danska J., Atkinson M.A. (2014). The intestinal microbiome in type 1 diabetes. Clin. Exp. Immunol..

[48] Kamada N., Seo S.-U., Chen G.Y., Núñez G. (2013). Role of the gut microbiota in immunity and inflammatory disease. Nat. Rev. Immunol..

[49] Janeway C. (1989). Approaching the Asymptote? Evolution and Revolution in Immunology. Cold Spring Harb. Symp. Quant. Biol..

[50] Akira S., Uematsu S., Takeuchi O. (2006). Pathogen recognition and innate immunity. Cell.

[51] Wen L., Ley R., Volchkov P.Y., Stranges P., Avanesyan L., Stonebraker A.C., Hu C., Wong F.S., Szot G.L., Bluestone J.A. (2008). Innate immunity and intestinal microbiota in the development of Type 1 diabetes. Nat. Cell Biol..

[52] Dewhirst F.E., Chien C.-C., Paster B.J., Ericson R.L., Orcutt R.P., Schauer D.B., Fox J.G. (1999). Phylogeny of the Defined Murine Microbiota: Altered Schaedler Flora. Appl. Environ. Microbiol..

[53] Paun A., Yau C., Danska J.S. (2017). The Influence of the Microbiome on Type 1 Diabetes. J. Immunol..

[54] Tai N., Peng J., Liu F., Gulden E., Hu Y., Zhang X., Chen L., Wong F.S., Ningwen T. (2016). Microbial antigen mimics activate diabetogenic CD8 T cells in NOD mice. J. Exp. Med..

[55] Nanjundappa R.H., Ronchi F., Wang J., Clemente-Casares X., Yamanouchi J., Umeshappa C.S., Yang Y., Blanco J., Bassolas-Molina H., Salas A. (2017). A Gut Microbial Mimic that Hijacks Diabetogenic Autoreactivity to Suppress Colitis. Cell.

[56] Gülden E., Chao C., Tai N., Pearson J., Peng J., Majewska-Szczepanik M., Zhou Z., Wong F.S., Wen L. (2018). TRIF deficiency protects non-obese diabetic mice from type 1 diabetes by modulating the gut microbiota and dendritic cells. J. Autoimmun..

[57] King C., Sarvetnick N. (2011). The Incidence of Type-1 Diabetes in NOD Mice Is Modulated by Restricted Flora Not Germ-Free Conditions. PLoS ONE.

[58] Kriegel M., Sefik E., Hill J.A., Wu H.-J., Benoist C., Mathis D. (2011). Naturally transmitted segmented filamentous bacteria segregate with diabetes protection in nonobese diabetic mice. Proc. Natl. Acad. Sci. USA.

[59] Hensgens M., Keessen E., Squire M., Riley T., Koene M., de Boer E., Lipman L., Kuijper E. (2012). Clostridium difficile infection in the community: A zoonotic disease?. Clin. Microbiol. Infect..

[60] Miranda M.C.G., Oliveira R.P., Torres L., Aguiar S.L.F., Pinheiro-Rosa N., Lemos L., Guimarães M.A., Reis D., Silveira T., Ferreira E. (2019). Frontline Science: Abnormalities in the gut mucosa of non-obese diabetic mice precede the onset of type 1 diabetes. J. Leukoc. Biol..

[61] Simon M.-C., Reinbeck A.L., Wessel C., Heindirk J., Jelenik T., Kaul K., Arreguin-Cano J., Strom A., Blaut M., Bäckhed F. (2020). Distinct alterations of gut morphology and microbiota characterize accelerated diabetes onset in nonobese diabetic mice. J. Biol. Chem..

[62] Mullaney J., Stephens J.E., Geeling B.E., Hamilton-Williams E.E. (2019). Early-life exposure to gut microbiota from disease-protected mice does not impact disease outcome in type 1 diabetes susceptible NOD mice. Immunol. Cell Biol..

[63] Hansen C.H.F., Krych L., Nielsen D.S., Vogensen F., Hansen L.H., Sørensen S., Buschard K., Hansen A.K. (2012). Early life treatment with vancomycin propagates Akkermansia muciniphila and reduces diabetes incidence in the NOD mouse. Diabetologia.

[64] Hu Y., Jin P., Peng J., Zhang X., Wong F.S., Wen L. (2016). Different immunological responses to early-life antibiotic exposure affecting autoimmune diabetes development in NOD mice. J. Autoimmun..

[65] Livanos A., Greiner T.U., Vangay P., Pathmasiri W., Stewart D., McRitchie S., Li H., Chung J., Sohn J., Kim S. (2016). Antibiotic-mediated gut microbiome perturbation accelerates development of type 1 diabetes in mice. Nat. Microbiol..

[66] Sofi M.H., Johnson B.M., Gudi R.R., Jolly A., Gaudreau M.-C., Vasu C. (2019). Polysaccharide A–Dependent Opposing Effects of Mucosal and Systemic Exposures to Human Gut CommensalBacteroides fragilisin Type 1 Diabetes. Diabetes.

[67] Joller N., Lozano E., Burkett P.R., Patel B., Xiao S., Zhu C., Xia J., Tan T.G., Sefik E., Yajnik V. (2014). Treg Cells Expressing the Coinhibitory Molecule TIGIT Selectively Inhibit Proinflammatory Th1 and Th17 Cell Responses. Immunity.

[68] Elhag D.A., Kumar M., Al Khodor S. (2020). Exploring the Triple Interaction between the Host Genome, the Epigenome, and the Gut Microbiome in Type 1 Diabetes. Int. J. Mol. Sci..

[69] Craig M.E., Kim K.W., Isaacs S., Penno M., Hamilton-Williams E., Couper J.J., Rawlinson W.D. (2019). Early-life factors contributing to type 1 diabetes. Diabetologia.

[70] Yamanouchi J., Rainbow D., Serra P., Howlett S., Hunter K., Garner V.E., Gonzalez-Munoz A., Clark J., Veijola R., Cubbon R. (2007). Interleukin-2 gene variation impairs regulatory T cell function and causes autoimmunity. Nat. Genet..

[71] Hunter K., Rainbow D., Plagnol V., Todd J.A., Peterson L.B., Wicker L.S. (2007). Interactions between Idd5.1/Ctla4 and other type 1 diabetes genes. J. Immunol..

[72] Mullaney J.A., Stephens J.E., Costello M.-E., Fong C., Geeling B.E., Gavin P., Wright C.M., Spector T.D., Brown M.A., Hamilton-Williams E.E. (2018). Type 1 diabetes susceptibility alleles are associated with distinct alterations in the gut microbiota. Microbiome.

[73] Thorburn A.N., Macia L., Mackay C.R. (2014). Diet, Metabolites, and “Western-Lifestyle” Inflammatory Diseases. Immunity.

[74] Huang J., Pearson J.A., Peng J., Hu Y., Sha S., Xing Y., Huang G., Li X., Hu F., Xie Z. (2020). Gut microbial metabolites alter IgA immunity in type 1 diabetes. JCI Insight.

[75] Yap Y.A., McLeod K.H., McKenzie C.I., Gavin P.G., Davalos-Salas M., Richards J.L., Moore R.J., Lockett T.J., Clarke J.M., Eng V.V. (2021). An acetate-yielding diet imprints an immune and anti-microbial programme against enteric infection. Clin. Transl. Immunol..

[76] Mariño E., Richards J.L., McLeod K.H., Stanley D., Yap Y.-A., Knight J., McKenzie C., Kranich J., Oliveira A.C., Rossello F.J. (2017). Gut microbial metabolites limit the frequency of autoimmune T cells and protect against type 1 diabetes. Nat. Immunol..

[77] Brauner H., Lüthje P., Grünler J., Ekberg N.R., Dallner G., Brismar K. (2014). Markers of innate immune activity in patients with type 1 and type 2 diabetes mellitus and the effect of the anti-oxidant coenzyme Q10 on inflammatory activity. Clin. Exp. Immunol..

[78] Sun J., Furio L., Mecheri R., van der Does A., Lundeberg E., Saveanu L., Chen Y., van Endert P., Agerberth B., Diana J. (2015). Pancreatic β-Cells Limit Autoimmune Diabetes via an Immunoregulatory Antimicrobial Peptide Expressed under the Influence of the Gut Microbiota. Immunity.

[79] Miani M., Le Naour J., Waeckel-Enée E., Verma S.C., Straube M., Emond P., Ryffel B., van Endert P., Sokol H., Diana J. (2018). Gut Microbiota-Stimulated Innate Lymphoid Cells Support β-Defensin 14 Expression in Pancreatic Endocrine Cells, Preventing Autoimmune Diabetes. Cell Metab..

[80] Warshauer J.T., Bluestone J.A., Anderson M.S. (2020). New Frontiers in the Treatment of Type 1 Diabetes. Cell Metab..

[81] Zhou H., Sun L., Zhang S., Zhao X., Gang X., Wang G. (2020). Evaluating the Causal Role of Gut Microbiota in Type 1 Diabetes and Its Possible Pathogenic Mechanisms. Front. Endocrinol..

[82] Al Theyab A., Almutairi T., Al-Suwaidi A.M., Bendriss G., McVeigh C., Chaari A. (2020). Epigenetic Effects of Gut Metabolites: Exploring the Path of Dietary Prevention of Type 1 Diabetes. Front. Nutr..

[83] de Goffau M., Luopajärvi K., Knip M., Ilonen J., Ruohtula T., Härkönen T., Orivuori L., Hakala S., Welling G.W., Harmsen H.J. (2013). Fecal Microbiota Composition Differs Between Children With -Cell Autoimmunity and Those Without. Diabetes.

[84] Lau K., Benitez P., Ardissone A., Wilson T.D., Collins E.L., Lorca G., Li N., Sankar D., Wasserfall C., Neu J. (2011). Inhibition of Type 1 Diabetes Correlated to a Lactobacillus johnsonii N6.2-Mediated Th17 Bias. J. Immunol..

[85] Calabrese M., Magliozzi R., Ciccarelli O., Geurts J.J.G., Reynolds R., Martin R. (2015). Exploring the origins of grey matter damage in multiple sclerosis. Nat. Rev. Neurosci..

[86] Glatigny S., Bettelli E. (2018). Experimental Autoimmune Encephalomyelitis (EAE) as Animal Models of Multiple Sclerosis (MS). Cold Spring Harb. Perspect. Med..

[87] Ransohoff R.M. (2012). Animal models of multiple sclerosis: The good, the bad and the bottom line. Nat. Neurosci..

[88] Tsunoda I., Fujinami R.S. (2009). Neuropathogenesis of Theiler’s Murine Encephalomyelitis Virus Infection, An Animal Model for Multiple Sclerosis. J. Neuroimmune Pharmacol..

[89] Pachner A.R. (2011). Experimental models of multiple sclerosis. Curr. Opin. Neurol..

[90] Dendrou C.A., Fugger L., Friese M.A. (2015). Immunopathology of multiple sclerosis. Nat. Rev. Immunol..

[91] Wlodarczyk A., Løbner M., Cédile O., Owens T. (2014). Comparison of microglia and infiltrating CD_11c_^+^ cells as antigen presenting cells for T cell proliferation and cytokine response. J. Neuroinflam..

[92] Codarri L., Gyülvészi G., Tosevski V., Hesske L., Fontana A., Magnenat L., Suter T., Becher B. (2011). RORγt drives production of the cytokine GM-CSF in helper T cells, which is essential for the effector phase of autoimmune neuroinflammation. Nat. Immunol..

[93] Grifka-Walk H.M., Giles D.A., Segal B.M. (2015). IL-12-polarized Th1 cells produce GM-CSF and induce EAE independent of IL-23. Eur. J. Immunol..

[94] Baecher-Allan C., Kaskow B., Weiner H.L. (2018). Multiple Sclerosis: Mechanisms and Immunotherapy. Neuron.

[95] Filippi M., Bar-Or A., Piehl F., Preziosa P., Solari A., Vukusic S., Rocca M.A. (2018). Multiple sclerosis. Nat. Rev. Dis. Prim..

[96] Ochoa-Repáraz J., Mielcarz D.W., Ditrio L.E., Burroughs A.R., Foureau D.M., Haque-Begum S., Kasper L.H. (2009). Role of Gut Commensal Microflora in the Development of Experimental Autoimmune Encephalomyelitis. J. Immunol..

[97] Lee Y.K., Menezes J.S., Umesaki Y., Mazmanian S.K. (2011). Proinflammatory T-cell responses to gut microbiota promote experimental autoimmune encephalomyelitis. Proc. Natl. Acad. Sci. USA.

[98] Diaz Heijtz R., Wang S., Anuar F., Qian Y., Björkholm B., Samuelsson A., Hibberd M.L., Forssberg H., Pettersson S. (2011). Normal gut microbiota modulates brain development and behavior. Proc. Natl. Acad. Sci. USA.

[99] Ang Q.Y., Alexander M., Newman J.C., Tian Y., Cai J., Upadhyay V., Turnbaugh J.A., Verdin E., Hall K.D., Leibel R.L. (2020). Ketogenic Diets Alter the Gut Microbiome Resulting in Decreased Intestinal Th17 Cells. Cell.

[100] Duc D., Vigne S., Bernier-Latmani J., Yersin Y., Ruiz F., Gaïa N., Leo S., Lazarevic V., Schrenzel J., Petrova T.V. (2019). Disrupting Myelin-Specific Th17 Cell Gut Homing Confers Protection in an Adoptive Transfer Experimental Autoimmune Encephalomyelitis. Cell Rep..

[101] Cosorich I., Dalla-Costa G., Sorini C., Ferrarese R., Messina M.J., Dolpady J., Radice E., Mariani A., Testoni P.A., Canducci F. (2017). High frequency of intestinal T H 17 cells correlates with microbiota alterations and disease activity in multiple sclerosis. Sci. Adv..

[102] Erny D., De Angelis A.L., Jaitin D., Wieghofer P., Staszewski O., David E., Keren-Shaul H., Mahlakoiv T., Jakobshagen K., Buch T. (2015). Host microbiota constantly control maturation and function of microglia in the CNS. Nat. Neurosci..

[103] Rothhammer V., Mascanfroni I.D., Bunse L., Takenaka M.C., Kenison J., Mayo L., Chao C.-C., Patel B., Yan R., Blain M. (2016). Type I interferons and microbial metabolites of tryptophan modulate astrocyte activity and central nervous system inflammation via the aryl hydrocarbon receptor. Nat. Med..

[104] Fan Y., Zhang J. (2019). Dietary Modulation of Intestinal Microbiota: Future Opportunities in Experimental Autoimmune Encephalomyelitis and Multiple Sclerosis. Front. Microbiol..

[105] Ii D.M.J., Goertz J.E., Marin I.A., Costello J., Overall C.C., Gaultier A. (2020). Experimental autoimmune encephalomyelitis is associated with changes of the microbiota composition in the gastrointestinal tract. Sci. Rep..

[106] Wilck N., Matus M.G., Kearney S.M., Olesen S.W., Forslund K., Bartolomaeus H., Haase S., Mähler A., Balogh A., Markó L. (2017). Salt-responsive gut commensal modulates TH17 axis and disease. Nature.

[107] Farez M.F., Fiol M.P., Gaitán M.I., Quintana F.J., Correale J. (2015). Sodium intake is associated with increased disease activity in multiple sclerosis. J. Neurol. Neurosurg. Psychiatry.

[108] Yamashita M., Ukibe K., Matsubara Y., Hosoya T., Sakai F., Kon S., Arima Y., Murakami M., Nakagawa H., Miyazaki T. (2018). Lactobacillus helveticus SBT2171 Attenuates Experimental Autoimmune Encephalomyelitis in Mice. Front. Microbiol..

[109] Montgomery T.L., Künstner A., Kennedy J.J., Fang Q., Asarian L., Culp-Hill R., D’Alessandro A., Teuscher C., Busch H., Krementsov D.N. (2020). Interactions between host genetics and gut microbiota determine susceptibility to CNS autoimmunity. Proc. Natl. Acad. Sci. USA.

[110] Miyauchi E., Kim S.-W., Suda W., Kawasumi M., Onawa S., Taguchi-Atarashi N., Morita H., Taylor T.D., Hattori M., Ohno H. (2020). Gut microorganisms act together to exacerbate inflammation in spinal cords. Nat. Cell Biol..

[111] Vamanu E., Rai S. (2021). The Link between Obesity, Microbiota Dysbiosis, and Neurodegenerative Pathogenesis. Diseases.

[112] Al-Assal K., Martinez A.C., Torrinhas R.S., Cardinelli C., Waitzberg D. (2018). Gut microbiota and obesity. Clin. Nutr. Exp..

[113] Ley R.E., Turnbaugh P.J., Klein S., Gordon J.I. (2006). Human gut microbes associated with obesity. Nature.

[114] Muscogiuri G., Cantone E., Cassarano S., Tuccinardi D., Barrea L., Savastano S., Colao A. (2019). Gut microbiota: A new path to treat obesity. Int. J. Obes. Suppl..

[115] Turnbaugh P.J., Hamady M., Yatsunenko T., Cantarel B.L., Duncan A., Ley R.E., Sogin M.L., Jones W.J., Roe B.A., Affourtit J.P. (2009). A core gut microbiome in obese and lean twins. Nature.

[116] Ji Z., Wu S., Xu Y., Qi J., Su X., Shen L. (2019). Obesity Promotes EAE Through IL-6 and CCL-2-Mediated T Cells Infiltration. Front. Immunol..

[117] Munger K.L., Chitnis T., Ascherio A. (2009). Body size and risk of MS in two cohorts of US women. Neurology.

[118] Hedström A.K., Olsson T., Alfredsson L. (2012). High body mass index before age 20 is associated with increased risk for multiple sclerosis in both men and women. Mult. Scler. J..

[119] Wesnes K., Riise T., Casetta I., Drulovic J., Granieri E., Holmøy T., Kampman M.T., Landtblom A.-M., Lauer K., Lossius A. (2014). Body size and the risk of multiple sclerosis in Norway and Italy: The EnvIMS study. Mult. Scler. J..

[120] Haghikia A., Jörg S., Duscha A., Berg J., Manzel A., Waschbisch A., Hammer A., Lee D.-H., May C., Wilck N. (2015). Dietary Fatty Acids Directly Impact Central Nervous System Autoimmunity via the Small Intestine. Immunity.

[121] Dalile B., Van Oudenhove L., Vervliet B., Verbeke K. (2019). The role of short-chain fatty acids in microbiota–gut–brain communication. Nat. Rev. Gastroenterol. Hepatol..

[122] Mizuno M., Noto D., Kaga N., Chiba A., Miyake S. (2017). The dual role of short fatty acid chains in the pathogenesis of autoimmune disease models. PLoS ONE.

[123] Aharoni R., Schottlender N., Bar-Lev D.D., Eilam R., Sela M., Tsoory M., Arnon R. (2019). Cognitive impairment in an animal model of multiple sclerosis and its amelioration by glatiramer acetate. Sci. Rep..

[124] Kim M., Lee S., Lim H., Lee J., Park J.-Y., Kwon H.-J., Lee I.-C., Ryu Y.-B., Kim J., Shin T. (2020). Oleanolic Acid Acetate Alleviates Symptoms of Experimental Autoimmune Encephalomyelitis in Mice by Regulating Toll-Like Receptor 2 Signaling. Front. Pharmacol..

[125] Chen T., Noto D., Hoshino Y., Mizuno M., Miyake S. (2019). Butyrate suppresses demyelination and enhances remyelination. J. Neuroinflam..

[126] Park J., Wang Q., Wu Q., Mao-Draayer Y., Kim C.H. (2019). Bidirectional regulatory potentials of short-chain fatty acids and their G-protein-coupled receptors in autoimmune neuroinflammation. Sci. Rep..

[127] Sonner J.K., Keil M., Falk-Paulsen M., Mishra N., Rehman A., Kramer M., Deumelandt K., Röwe J., Sanghvi K., Wolf L. (2019). Dietary tryptophan links encephalogenicity of autoreactive T cells with gut microbial ecology. Nat. Commun..

[128] Schmidt T., Raes J., Bork P. (2018). The Human Gut Microbiome: From Association to Modulation. Cell.

[129] Berer K., Gerdes L.A., Cekanaviciute E., Jia X., Xiao L., Xia Z., Liu C., Klotz L., Stauffer U., Baranzini S. (2017). Gut microbiota from multiple sclerosis patients enables spontaneous autoimmune encephalomyelitis in mice. Proc. Natl. Acad. Sci. USA.

[130] Mangalam A., Shahi S.K., Luckey D., Karau M., Marietta E., Luo N., Choung R.S., Ju J., Sompallae R., Gibson-Corley K. (2017). Human Gut-Derived Commensal Bacteria Suppress CNS Inflammatory and Demyelinating Disease. Cell Rep..

[131] Borody T., Leis S., Campbell J., Torres M., Nowak A. (2011). Fecal Microbiota Transplantation (FMT) in Multiple Sclerosis (MS). Am. J. Gastroenterol..

[132] Makkawi S., Camara-Lemarroy C., Metz L. (2018). Fecal microbiota transplantation associated with 10 years of stability in a patient with SPMS. Neurol.-Neuroimmunol. Neuroinflamm..

[133] Junker A., Hohlfeld R., Meinl E. (2010). The emerging role of microRNAs in multiple sclerosis. Nat. Rev. Neurol..

[134] Juźwik C.A., Drake S., Lécuyer M.-A., Johnson R.M., Morquette B., Zhang Y., Charabati M., Sagan S.M., Bar-Or A., Prat A. (2018). Neuronal microRNA regulation in Experimental Autoimmune Encephalomyelitis. Sci. Rep..

[135] Liu S., Rezende R.M., Moreira T.G., Tankou S.K., Cox L.M., Wu M., Song A., Dhang F.H., Wei Z., Costamagna G. (2019). Oral Administration of miR-30d from Feces of MS Patients Suppresses MS-like Symptoms in Mice by Expanding Akkermansia muciniphila. Cell Host Microbe.

[136] McInnes I.B., Schett G. (2011). The pathogenesis of rheumatoid arthritis. N. Engl. J. Med..

[137] Smolen J.S., Aletaha D., McInnes I.B. (2016). Rheumatoid arthritis. Lancet.

[138] Karpouzas G.A., Ormseth S.R., Hernandez E., Budoff M.J. (2019). Impact of Cumulative Inflammation, Cardiac Risk Factors, and Medication Exposure on Coronary Atherosclerosis Progression in Rheumatoid Arthritis. Arthritis Rheumatol..

[139] Taneja V. (2015). Cytokines pre-determined by genetic factors are involved in pathogenesis of Rheumatoid arthritis. Cytokine.

[140] Sugiyama D., Nishimura K., Tamaki K., Tsuji G., Nakazawa T., Morinobu A., Kumagai S. (2009). Impact of smoking as a risk factor for developing rheumatoid arthritis: A meta-analysis of observational studies. Ann. Rheum. Dis..

[141] Wells P.M., Williams F.M., Matey-Hernandez M., Menni C., Steves C. (2019). ‘RA and the microbiome: Do host genetic factors provide the link?. J. Autoimmun..

[142] MacGregor A.J., Snieder H., Rigby A.S., Koskenvuo M., Kaprio J., Aho K., Silman A.J. (2000). Characterizing the quantitative genetic contribution to rheumatoid arthritis using data from twins. Arthritis Rheumatol..

[143] Maeda Y., Kurakawa T., Umemoto E., Motooka D., Ito Y., Gotoh K., Hirota K., Matsushita M., Furuta Y., Narazaki M. (2016). Dysbiosis Contributes to Arthritis Development via Activation of Autoreactive T Cells in the Intestine. Arthritis Rheumatol..

[144] Sakaguchi N., Takahashi T., Hata H., Nomura T., Tagami T., Yamazaki S., Sakihama T., Matsutani T., Negishi I., Nakatsuru S. (2003). Altered thymic T-cell selection due to a mutation of the ZAP-70 gene causes autoimmune arthritis in mice. Nat. Cell Biol..

[145] Rogier R., Ederveen T.H.A., Boekhorst J., Wopereis H., Scher J.U., Manasson J., Frambach S.J.C.M., Knol J., Garssen J., Van Der Kraan P.M. (2017). Aberrant intestinal microbiota due to IL-1 receptor antagonist deficiency promotes IL-17- and TLR4-dependent arthritis. Microbiome.

[146] Abdollahi-Roodsaz S., Joosten L.A., Koenders M., Devesa I., Roelofs M.F., Radstake T.R., Heuvelmans-Jacobs M., Akira S., Nicklin M., Ribeiro-Dias F. (2008). Stimulation of TLR2 and TLR4 differentially skews the balance of T cells in a mouse model of arthritis. J. Clin. Investig..

[147] Rehaume L.M., Mondot S., de Cárcer D.A., Velasco J., Benham H., Hasnain S., Bowman J., Ruutu M., Hansbro P., McGuckin M. (2014). ZAP-70 Genotype Disrupts the Relationship Between Microbiota and Host, Leading to Spondyloarthritis and Ileitis in SKG Mice. Arthritis Rheumatol..

[148] Jubair W.K., Hendrickson J.D., Severs E.L., Schulz H.M., Adhikari S., Ir D., Pagan J.D., Anthony R.M., Robertson C.E., Frank D.N. (2018). Modulation of Inflammatory Arthritis in Mice by Gut Microbiota Through Mucosal Inflammation and Autoantibody Generation. Arthritis Rheumatol..

[149] Espina M.D.T., Gabarrini G., Harmsen H.J.M., Westra J., Van Winkelhoff A.J., Van Dijl J.M. (2019). Talk to your gut: The oral-gut microbiome axis and its immunomodulatory role in the etiology of rheumatoid arthritis. FEMS Microbiol. Rev..

[150] Chu X.-J., Cao N.-W., Zhou H.-Y., Meng X., Guo B., Zhang H.-Y., Li B.-Z. (2021). The oral and gut microbiome in rheumatoid arthritis patients: A systematic review. Rheumatology.

[151] Vaahtovuo J., Munukka E., Korkeamäki M., Luukkainen R., Toivanen P. (2008). Fecal microbiota in early rheumatoid arthritis. J. Rheumatol..

[152] Scher J.U., Sczesnak A., Longman R.S., Segata N., Ubeda C., Bielski C., Rostron T., Cerundolo V., Pamer E.G., Abramson S.B. (2013). Expansion of intestinal Prevotella copri correlates with enhanced susceptibility to arthritis. eLife.

[153] Li Y., Zhang S.-X., Yin X.-F., Zhang M.-X., Qiao J., Xin X.-H., Chang M.-J., Gao C., Li Y.-F., Li X.-F. (2021). The Gut Microbiota and Its Relevance to Peripheral Lymphocyte Subpopulations and Cytokines in Patients with Rheumatoid Arthritis. J. Immunol. Res..

[154] Wells P.M., Adebayo A.S., Bowyer R.C.E., Freidin M.B., Finckh A., Strowig T., Lesker T.R., Alpizar-Rodriguez D., Gilbert B., Kirkham B. (2020). Associations between gut microbiota and genetic risk for rheumatoid arthritis in the absence of disease: A cross-sectional study. Lancet Rheumatol..

[155] Alpízar-Rodríguez D., Finckh A., Gilbert B. (2020). The Role of Nutritional Factors and Intestinal Microbiota in Rheumatoid Arthritis Development. Nutrients.

[156] Gan R.W., Demoruelle M.K., Deane K.D., Weisman M.H., Buckner J.H., Gregersen P.K., Mikuls T.R., O’Dell J.R., Keating R.M., Fingerlin T.E. (2017). Omega-3 fatty acids are associated with a lower prevalence of autoantibodies in shared epitope-positive subjects at risk for rheumatoid arthritis. Ann. Rheum. Dis..

[157] Di Giuseppe D., Crippa A., Orsini N., Wolk A. (2014). Fish consumption and risk of rheumatoid arthritis: A dose-response meta-analysis. Arthritis Res. Ther..

[158] Venkatraman B.S.R.A. (2000). Increased Permeability in Dextran Sulphate Colitis in Rats: Time Course of Development and Effect of Butyrate. Scand. J. Gastroenterol..

[159] Tajik N., Frech M., Schulz O., Schälter F., Lucas S., Azizov V., Dürholz K., Steffen F., Omata Y., Rings A. (2020). Targeting zonulin and intestinal epithelial barrier function to prevent onset of arthritis. Nat. Commun..

[160] Wu W., Sun M., Chen F., Cao A.T., Liu H., Zhao Y., Huang X., Xiao Y., Yao S., Zhao Q. (2017). Microbiota metabolite short-chain fatty acid acetate promotes intestinal IgA response to microbiota which is mediated by GPR43. Mucosal Immunol..

[161] Fasano A., Not T., Wang W., Uzzau S., Berti I., Tommasini A., Goldblum S.E. (2000). Zonulin, a newly discovered modulator of intestinal permeability, and its expression in coeliac disease. Lancet.

[162] Häger J., Bang H., Hagen M., Frech M., Träger P., Sokolova M., Steffen U., Tascilar K., Sarter K., Schett G. (2019). The Role of Dietary Fiber in Rheumatoid Arthritis Patients: A Feasibility Study. Nutrients.

